# The Role of KLF_4_ in Alzheimer’s Disease

**DOI:** 10.3389/fncel.2018.00325

**Published:** 2018-09-21

**Authors:** Ziqian Cheng, Xiaohan Zou, Yang Jin, Shuohui Gao, Jiayin Lv, Bingjin Li, Ranji Cui

**Affiliations:** ^1^Jilin Provincial Key Laboratory on Molecular and Chemical Genetic, The Second Hospital of Jilin University, Changchun, China; ^2^Department of Orthopedics, China-Japan Union Hospital of Jilin University, Changchun, China; ^3^Department of Gastrointestinal Colorectal Surgery, China-Japan Union Hospital of Jilin University, Changchun, China

**Keywords:** Alzheimer’s disease, KLF_4_, stem cell, neuroinflammation, apoptosis

## Abstract

Krüppel-like factor 4 (KLF_4_), a member of the family of zinc-finger transcription factors, is widely expressed in range of tissues that play multiple functions. Emerging evidence suggest KLF_4_’s critical regulatory effect on the neurophysiological and neuropathological processes of Alzheimer’s disease (AD), indicating that KLF_4_ might be a potential therapeutic target of neurodegenerative diseases. In this review, we will summarize relevant studies and illuminate the regulatory role of KLF_4_ in the neuroinflammation, neuronal apoptosis, axon regeneration and iron accumulation to clarify KLF_4_’s status in the pathogenesis of AD.

## Introduction

Kruppel-like factor 4 (KLF_4_) is a member of the family of zinc-finger transcription factor, which is expressed in various human tissues. It is well known as one of the four factors of the induction to pluripotent stem cells (iPSCs) (Ghaleb and Yang, 2017). KLF_4_ can regulate multiple important biological processes such as neuroinflammation, oxidative stress, proliferation, differentiation, and apoptosis (Kaushik et al., 2010; Mamonkin et al., 2013; Zhang et al., 2015; Miao et al., 2017; Xu et al., 2017). Amounts of previous studies focused on KLF_4_’s role in cancer development and progression (Karam et al., 2017; Yadav et al., 2018). KLF_4_ is a dual-function transcription factor, which can exert its role as an oncogene or a tumor suppressor gene depending on the cancer type or cancer stage (Evans and Liu, 2008). It can activate or inhibit transcription of genes involved in cell proliferation, differentiation and apoptosis (Ding et al., 2015). KLF_4_ can collaborate with other reprogramming factors to convert the somatic cells into iPSCs and inhibit the differentiation of stem cells (Takahashi and Yamanaka, 2006; van Schaijik et al., 2018). This provides therapeutic prospects for vascular diseases, immune diseases, anorexia and other diseases (Imbernon et al., 2014; Liu Y. et al., 2015; Murgai et al., 2017). Moreover, KLF_4_ can play a widely regulatory role in the central nervous system (CNS). Several studies indicate that KLF_4_ is linked to multiple neurological disorders, including Alzheimer’s disease (AD), epilepsy, Parkinson’s disease, hydrocephalus and schizophrenia (Qin et al., 2011; Xie et al., 2013; Han et al., 2015; Nishiguchi et al., 2015; Li L. et al., 2017).

AD is one of the most common chronic neurodegenerative diseases, which leads to cognitive and memory impairments, various mental symptoms and behavioral abnormality and progressive dementia is the most common clinical feature (Jiang et al., 2018). The current confirmed pathogenic factors of AD include the formation of senile plaques induced by abnormal amyloid-β (Aβ) deposition and the neurofibrillary tangles or dystrophic neuritis induced by tau accumulation (Querfurth and LaFerla, 2010; Shinohara et al., 2014). In addition, AD can be also affected by genetic factors. However, the elicit pathogenesis is still obscure. The most prevalent drugs for AD treatment include neurotransmitter enhancers, anti-Amyloid agents, neuroprotective peptides, and other drugs (Cacabelos, 2018). Notably, several studies have showed that KLF_4_ played a significant role in the pathogenesis of AD. In this review, we focus on the regulatory role of KLF_4_ in neuroinflammation, neuronal apoptosis, axonal regeneration, and iron accumulation to explain the association between KLF_4_ and the pathogenesis of AD, which might provide insights into the cellular and molecular mechanisms of neurodegenerative disorders.

## The Biological Characteristics of KLF_4_

KLF_4_ is a zinc finger-containing nuclear protein, isolated from NIH 3T3 library and located in the cell nucleus. It was first identified and characterized by Shields et al. (1996). The molecular mass of human KLF_4_ is 55kD and it is located on the chromosome 9q31. KLF_4_ covers a 6.3 kb gene segment and has five exons. Its cDNA coding region encodes a polypeptide consisting of 470 amino acid residues (Yet et al., 1998; Ghaleb and Yang, 2017). The carboxy terminus of KLF_4_ has a DNA binding structure region containing three Cys2His2 (C2H2) type zinc finger structures, which are formed by 81 highly conserved amino acids. It regulates transcription by high affinity with CACCC elements and GC-rich target gene DNA sequences (Shields and Yang, 1998; Pearson et al., 2008). Most of the DNA-binding sites of KLF_4_ are located within the zinc finger region, including N-terminal transcription activation domain for proteins interacting, C-terminal zinc finger structure for DNA binding and transcription inhibition zone (Bieker, 2001). KLF_4_ is involved in regulating the expression of many endogenous genes (Shields and Yang, 1998). There is a highly variable transcriptional regulatory domain at the amino terminus of KLF_4_. The amino acid residues located between the 91 and the 117 amino constitute a transcriptional activation domain, which is rich in proline and serine, while a transcriptional repression domain also exists. Therefore, KLF_4_ has two adverse effects: activating and inhibiting gene transcription (Yet et al., 1998; Wei et al., 2006).

During the embryonic development, KLF_4_ was higher expressed in the late stage of embryonic development. While in mature tissues and organs, KLF_4_ is mainly expressed in the gastrointestinal tract, oral cavity, skin epidermis, vascular endothelium and kidney, and is less expressed in the brain (Segre et al., 1999; Ghaleb et al., 2011; Liu et al., 2013; Chen et al., 2015; He et al., 2015; Bin et al., 2016). It is thought to play significant role in regulating cell proliferation and differentiation. Besides, KLF_4_ can also regulate cell cycle. KLF_4_ can activate P21 in a P53-dependent manner (Zhang et al., 2000). In addition, It was found that KLF_4_ (–/–) cells entered senescence phase earlier than KLF_4_ (+/+) cells, which can be explained by the less antioxidant gene expression and higher reactive oxygen species (ROS) level in KLF_4_ (–/–) cells. ROS can increase p53 and p21 expression and subsequently promote the DNA damage (Liu C. et al., 2015). It was found that PRMT5 can elevate the KLF_4_ expression in protein levels. PRMT5 was reported to increase the transcription of p21 and decrease the expression of bax via inhibiting KLF_4_ ubiquitylation (Hu et al., 2015). Furthermore, numerous studies have demonstrated that KLF_4_ is involved in regulation of apoptosis of neurons (Kong et al., 2016; Cui et al., 2017; Song et al., 2018). Physiological regulatory role of KLF_4_ that we have known are still little and further investigations are needed.

## Role of KLF_4_ in AD

It is well established that AD is mainly characterized by memory and cognitive impairments and executive dysfunction (Goedert and Spillantini, 2006). Many studies have demonstrated that neuronal apoptosis and synaptic dysfunction are pathological basis of the decline of cognitive function (Caccamo et al., 2017; Guo et al., 2017; Yoon et al., 2018). The accumulated damage of Aβ deposition, oxidative stress and iron accumulation can lead to neuronal dysfunction and apoptosis of AD patients. Several studies have shown that KLF_4_’s regulatory role appears to be crucial in CNS. Considering that KLF_4_ was reported to regulate neuronal apoptosis, synaptic regeneration, oxidative stress and neuroinflammation, the relationship between KLF_4_ and the pathogenesis of AD might be a potential novel target for AD treatment.

### Role of KLF_4_ in Neuroinflammation

Amounts of clinical studies have shown that Aβ can aggregate and is the main component of the extracellular deposits of the brain tissue of AD patients, which can impair the surrounding synapses and neurons, and lead to neuronal death. Abnormal secretion or excessive production of Aβ leads to pathological changes of AD, so Aβ deposition is the core link of AD (Rajmohan and Reddy, 2017). In addition, studies have shown that excessive Aβ deposition can stimulate glial cells to secrete ROS and other influencing factors, leading to oxidative stress. It was known that oxidative stress can stimulate the production of Aβ. Therefore, Aβ and oxidative stress can interact with each other and affect the progression of AD (Cheignon et al., 2018).

**FIGURE 1:**
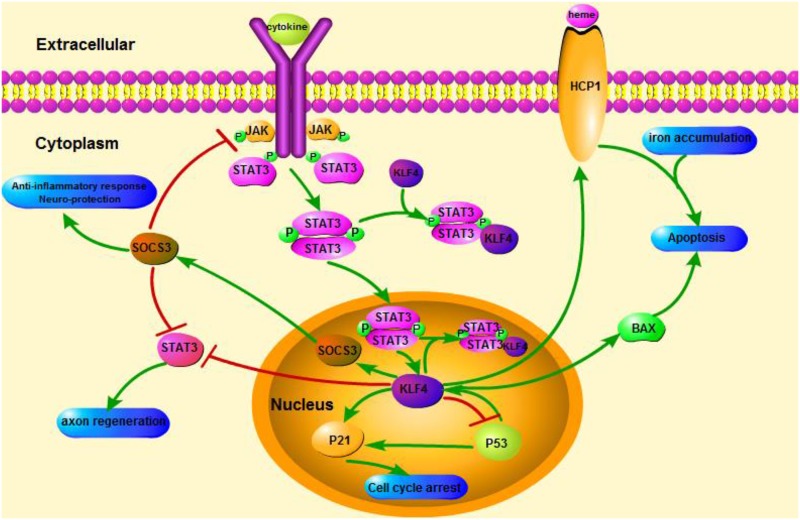
Schematicillustration of KLF_4_ related signaling pathways. This figure highlights the role of KLF_4_ in neuroprotection and axon regeneration. The arrows in the figure indicate activation or promotion, and the straight lines indicate related inhibition. KLF_4_, Kruppel-like factor 4; STAT3, Signal transducer and activator of transcription 3; JAK, Janus Kinase; SOCS3, Suppressor of cytokine signaling 3; HCP1, heme carrier protein 1; ERK5, mitogen-activated protein [MAP] kinase 5.

KLF_4_ was reported as a potential modulator and has a great effect on inflammation by mediating macrophages and endothelial cells (**Figure 1**) (Yoshida et al., 2014; Kapoor et al., 2015; Yang et al., 2018). In the CNS, excessive and chronic inflammatory reactions can cause damage of neuron and neurogliocyte. It was recently demonstrated that the KLF_4_ expression positively correlated with Aβ42-induced neuroinflammation. In microglial BV2 cells, oligomeric Aβ42 can increase KLF_4_ expression, which is mediated by activated P53 (Li L. et al., 2017). Under inflammatory conditions, such as Aβ accumulation, the release of pro-inflammatory cytokines may be stimulated in the generation of AD (Griffin and Barger, 2010). Neurotoxicity potency and pro-inflammatory potency of soluble Aβ42 oligomers is relatively higher than insoluble fiber deposit (Selkoe, 1991; Weinberg et al., 2018). Silence of KLF_4_ is able to restore Aβ42-mediated neuroinflammation, and overexpression of KLF_4_ can exacerbate Aβ42-mediated neuroinflammation (Li L. et al., 2017). Aβ accumulation induces activation of astrocytes and microglia (Rodríguez et al., 2016). Activated astrocytes can enhance the neuroinflammation by releasing pro-inflammatory factors such as IL-1, IL-6, and TNF-α (Rubio-Perez and Morillas-Ruiz, 2012; Doméné et al., 2016). The vicious cycle of inflammatory responses eventually leads to dysfunction and neuronal apoptosis.

KLF4 plays a crucial role in regulating pro-inflammatory signals. In glial cells, gemfibrozil-induced KLF4 activation increases suppressor of cytokine signal 3 (SOCS3) via PI3-kinase-AKT pathway (Ghosh and Pahan, 2012). The SiRNA-mediated knockdown of KLF4 could attenuate the level of SOCS in astroglia and microglia of mice, which could subsequently affect the expression of inflammatory gene (Kaushik et al., 2010; Ghosh and Pahan, 2012). In addition, SOCS deletion can promote the survival of injured neurons and promote axon regeneration (Smith et al., 2009; Sun et al., 2011). And KLF_4_ positively regulates the production of IL-1β or other pro-inflammatory markers. It positively regulates cyclooxygenase-2 (Cox-2) and negatively regulates inducible nitric oxide synthase (iNOS) (Kaushik et al., 2013). In addition, KLF_4_ is an important regulatory factor for monocyte differentiation and a potential target for immune regulation (Alder et al., 2008). Therefore, KLF_4_ might promote neuroinflammation by regulating these negative regulators.

It is worth mentioning that in Parkinson’s disease model, KLF_4_ can promote MPP+-induced oxidative stress and neurotoxicity, and then increase neuronal apoptosis and delay the cell proliferation (Chen et al., 2013). Oxidative stress is an imbalance between peroxidation and antioxidation. Free radicals can cause changes in different macromolecules, leading to cell damage, cell aging and tissue damage (Parajuli et al., 2013; Nie et al., 2015). Oxidative stress can aggravate early inflammation and Aβ production and then aggravate AD (Cai et al., 2011). Therefore, KLF_4_ may be involved in oxidative stress in AD.

These findings imply KLF_4_ a key role in mediating neuroinflammation by activating the microglia and the consequently release of pro-inflammatory cytokines. It has potential to enhance neuroinflammation. So far, many studies on the pathogenesis of AD have focused on neuroinflammation. As a potential target for immune regulation, KLF_4_ can promote the inflammatory responses of microglia via affecting related negative regulators, which has a great effect on the development of AD.

### Role of KLF_4_ in Apoptosis

Neurodegenerative changes include gradual loss of neurons and synapses in the representative brain regions, such as the cerebral cortex, hippocampus and other subcortical regions. The functional impairments of CNS induced by neuronal loss are permanent (Citron, 2010). Sustained oxidative stress can lead to neuronal apoptosis (Wu et al., 2010). A large number of studies have confirmed that AD is closely related to oxidative stress (Lee et al., 2012; Yui et al., 2015). It was found that chronic oxidative stress can enhance the expression of Phospholipase A2 group 3 (Pla2g3) in astrocytes and disrupt the balance of Aβ, and consequently lead to the development of AD (Yui et al., 2015).

Many studies have demonstrated that KLF_4_ plays an important role in inhibiting the development of oxidative stress (Shi et al., 2014; Liu C. et al., 2015). It was found that KLF_4_ can promote the cells apoptosis induced by H_2_O_2_, this action is likely to be caused by increased bax expression and decreased bcl-2 expression (Li et al., 2010). Quercetin could reduce KLF_4_ expression in human neuroblastoma SH-SY5Y cells, and increase the expression of bcl-2/bax ratio. Furthermore, Quercetin can moderate the apoptosis rate of SH-5YSY cell and reduce caspase-3 enzyme activity (Xi et al., 2012). A recent study investigated the neuroprotective effect of mitogen-activated protein (MAP) kinase 5 (ERK5) against oxidative stress. Activation of ERK5 can partially reduce H_2_O_2_-induced hippocampal neurons death and increase the NGF- and PC-induced neuroprotection (Su et al., 2014). Nils et al. used a mutant of MEK5 (MEK5D) to study the ERK5-activated transcription and functional responses in human endothelial cells, and identified KLF_4_ was a novel downstream ERK5 target (Ohnesorge et al., 2010). It was found that overexpression of KLF_4_ can suppress TNF-mediated inflammatory responses and reduce leukocyte adhesion and basal cell apoptosis. These results confirm that KLF_4_ has anti-inflammatory and anti-apoptotic properties (Ohnesorge et al., 2010). Subsequent experiments have demonstrated that the disappearance of cerebral cavernous malformation 1 (CCM1) in endothelial cells activates ERK5 via MEKK3-MEK5 signal pathway and increases KLF_4_ expression (Cuttano et al., 2016). ERK5 plays a mediating role in preconditioning (PC) and nerve growth factor (NGF) up-regulated the expression of KLF_4_ (Su et al., 2014). In addition, RNAi-mediated knocking-down of KLF_4_ can also reduce NGF- or PC-induced neuroprotection. Overexpression of KLF_4_ leads to higher bcl-2/bax ratio in H2O2-stressed cells (Su et al., 2014). Over-expressed KLF_4_ accelerates changes in bcl-2 and bax by combining with its corresponding promoter (Li et al., 2010). ERK5/KLF_4_ cascade may act as a pivot in various pathways which protect neurons from oxidative stress-induced death (Su et al., 2014).

Oxidative stress has been considered to be closely related to many degenerative diseases. KLF_4_ plays significant roles in maintaining genomic stability in oxidative stress. KLF_4_ and ERK5 act together to protect neurons from oxidative stress-induced apoptosis. Therefore, KLF_4_ may act as a therapeutic target to act against oxidative stress when it activated. It has been reported that statin drugs can activate ERK5, leading to the expression of KLF_4_ and its dependent genes (Ohnesorge et al., 2010), but the mechanism remains unclear, and KLF_4_ related upstream and downstream target genes are less studied in oxidative stress, there is a need for further study.

### Role of KLF_4_ in Axon Regeneration

Early axon loss is a common feature of neurodegenerative diseases. Synaptic loss and transport impairment in AD can cause cognitive impairments (Holtzman et al., 2011; Coleman, 2013). The degree of declarative memory damage is related to the synaptic density in the hippocampus and cortex. Soluble Aβ oligomers reduce glutamate uptake and promote synaptic dysfunction, disrupting synaptic plasticity (Li et al., 2009). Therefore, it is particularly important to study how to repair the axons in the CNS. In retinal ganglion cells, axons have a strong ability to grow and regenerate during early development, but in the CNS of adult mammals, axons lose their regeneration capacity and the neurons may graduate to die or atrophy (Goldberg and Barres, 2000; Goldberg et al., 2002).

KLF_4_ plays an important role in inhibiting axon growth. In embryonic RGCs, overexpression of KLF_4_ can reduce the percentage of neurite elongation, the length of axons and dendrites, and the neurite branching. Besides, it was found that the overexpression of KLF_4_ can reduce long-term postnatal axon growth rates but failed to reduce short-term axon growth rates (Moore et al., 2009; Steketee et al., 2014). Later studies have found that the axon bundles of KLF_4_–cKO mice were thicker than control mice (Fang et al., 2016). In addition, removal of KLF_4_ expression during development can increase the reproductive potential of adult RGCs. In addition, KLF_4_ lacking the c-terminal DNA binding domain had no effect on the axon growth. There was no impact on the survival of cells after retinal ganglion cells were injured if the KLF_4_ was knocking-out (Moore et al., 2009).

KLF_4_ can also affect the axonal regeneration. A recent study reported that the decrease of KLF_4_ expression in adult retinal ganglion cells promoted axon regeneration through JAK-STAT3 pathway (Qin et al., 2013). KLF_4_ increased the phosphorylation of STAT3, and regulated the axon growth via JAK-STAT signaling (Qin and Zhang, 2012). Under the treatment of cytokines, members of STAT family of proteins are phosphorylated at the carboxy-terminal tyrosine and serine sites within the cell to form a stable dimer. This modification enhances transcription of cell-associated genes (Yuan et al., 2005). The interaction between KLF_4_ and STAT3 on cytokine-induced phosphorylation of tyrosin705 inhibits the expression of STAT3 by inhibiting the binding of STAT3 to DNA (Qin et al., 2013). KLF_4_ knockdown obviously improves axon’s regeneration in retinal ganglion cells after injury of optic nerve, and prevents the nerve from injury after mild brain injury. The actions are mediated by a decrease in p-p53 and an increase in pSTAT3 levels. KLF_4_ positively regulates neuronal apoptosis via the p53 and JAK-STAT3 pathways, and KLF_4_ negatively regulates axonal repair via the JAK-STAT3 pathway (Cui et al., 2017).

Therefore, we hypothesized that in AD, axonal regeneration can be accomplished by altering the expression of KLF_4_ or altering intracellular related signaling pathways, and controlling AD progression by reducing missing axons or reducing axonal dysfunction. However, how to use the KLF_4_ transcription factor in potential therapeutics still needs further exploration.

### Role of KLF_4_ in Iron Accumulation

Iron is widely found in biological systems, iron-related metalloproteinases play a key role in transporting oxygen, transferring electrons, and catalyzing biochemical reactions (Aisen et al., 2001). However, any excess of iron beyond the normal physiological range can damage human health (Adlard and Bush, 2006). Studies have found that iron content in the hippocampus is negatively correlated with the performance of memory tests (Ding et al., 2009). Increased iron load in the brain accelerates the formation of Aβ plaques and hyperphosphorylated tau tangles, while also enhancing oxidative stress (Peters et al., 2015). Iron, which has a high degree of permeability, promotes nerve growth and cell-to-cell connections during brain development (Dallman and Spirito, 1977).

A recent study demonstrated that physiological stress caused activation of the KLF_4_-HCP1 signaling pathway and increased heme uptake (Li H. et al., 2017). Heme accounts for 95% of the functional iron in the human body. It is one of the main components of heme oxygenase (Hooda et al., 2014; Kurucz et al., 2018). Increasing the activity of oxygenase-1 can delay oxidation of the aging brain (Verdile et al., 2015; Serini and Calviello, 2016; Kurucz et al., 2018). This has a relief effect on AD. Physiological stress induces glucocorticoid level rise, glucocorticoid increases heme carrier protein 1 (HCP1) expression via KLF_4_, and then HCP1 promotes heme uptake (Li H. et al., 2017). Glucocorticoid and KLF_4_ regulate anti-inflammatory genes together, and cells with low glucocorticoid content cannot fully induce KLF_4_ expression (Sevilla et al., 2015). KLF_4_-induced increase in heme intake leads to iron accumulation in the brain. Iron promotes the release of ROS (Tronel et al., 2013). Iron element enhances brain oxidative stress in rats under psychological stress (Yu et al., 2011). Therefore, HCP1 may be regulated by KLF_4_ and glucocorticoid together. Increasing HCP1 enhances heme uptake, which leads directly to iron accumulation in the brain, exacerbates oxidation, increases apoptosis or dysfunction and worsens brain damage.

It is generally accepted that the memory and learning disability are the main symptoms of AD. A large number of clinical data have shown that Aβ plaque load and iron accumulation response to the development of learning and cognitive dysfunction in AD (van Bergen et al., 2018). Recently published data has suggested that, high-dose iron increases Aβ deposition and attenuates learning and memory in mice (Guo et al., 2013). Clinical studies have shown that iron-containing microglia is found in the hippocampus of AD patients under magnetic resonance imaging (Zeineh et al., 2015). Microglia acquires iron from transferring or non-transferring, extracellular and intracellular sources (McCarthy et al., 2018). Selective and sustained KLF_4_ expression can be induced in the nucleus and cytoplasm of ischemic hippocampal reactive astrocytes (Park et al., 2014). Studies have shown that KLF_4_ acts as a transcriptional repressor. It down-regulates the expression of ELK-3, and then ELK-3 inhibits the expression of HO-1 (Tsoyi et al., 2015). Heme oxygenase-1 (HO-1) is a stress protein that degrades heme into bilirubin, free iron, and carbon monoxide. Up-regulation of HO-1 in astrocytes can lead to abnormal iron deposition and mitochondrial dysfunction in the brain, leading to decreased cognitive ability (Schipper, 1999, 2004). Therefore, KLF_4_ may be involved in the process of iron accumulation in astrocytes, exacerbating oxidation in AD and aggravating brain damage.

## Conclusion

KLF_4_ is commonly known to play a pivotal role in regulating cell proliferation, apoptosis, and differentiation. Previous studies have focused on the regulation of KLF_4_ in several important neurophysiological processes, including neuroinflammation, neuroprotection and synaptic regeneration. Recently, KLF_4_ has been found to play an important role in the pathogenesis of AD. In this article, we review the role of KLF_4_ in neuroprotection and neurogenesis in AD.

KLF_4_ is not only a regulator of regulation of cell proliferation and differentiation, but also a potential target for regulating immune responses. KLF_4_ may regulate negative inflammatory factors and promote inflammatory response, and have a great effect on the expression of astrocyte nuclear microglia. In addition, KLF_4_ and ERK5 can act together to exert neuroprotective actions. Furthermore, axon regeneration can be accomplished by altering the content of specific transcription factors, intracellular inhibitors, or altering intracellular signaling pathways. Knocking out KLF_4_ can enhance the axon regeneration and accelerate axon growth rate. Reduction of KLF_4_ expression promotes axon regeneration through the JAK-STAT3 pathway, and KLF_4_ promotes the JAK-STAT3 pathway to further axon regeneration. Therefore, KLF_4_ might be involved in the process of anti-inflammatory, anti-apoptosis, axon regeneration and iron accumulation in the CNS, which plays a pivotal role in the AD generation. These findings suggest that KLF_4_ represents a potential therapeutic target for AD. However, the deep cellular and molecular mechanisms of the effects of KLF_4_ on AD remain unclear and further investigations are needed.

## Author Contributions

ZQC, XHZ, and YJ wrote the manuscript. SHG and JYL modified the framework of the manuscript. BJL and RJC provided the critical revisions. All authors approved the final version of the manuscript for submission.

## Conflict of Interest Statement

The authors declare that the research was conducted in the absence of any commercial or financial relationships that could be construed as a potential conflict of interest.
